# Increased Production of Angiopoietin-like Protein 2 in a Ligature- and LPS-Induced Periodontitis Mouse Model May Promote Colorectal Tumor Progression

**DOI:** 10.3390/jcm15062359

**Published:** 2026-03-19

**Authors:** Mika Yamashita, Genta Yamamoto, Kodai Katsumata, Daiki Takeuchi, Nayu Tachikawa, Kota Ono, Tasuku Ohno, Eisaku Nishida, Tsuyoshi Fujita, Jun-Ichiro Hayashi, Takeshi Kikuchi, Yoshihiko Sugita, Akio Mitani

**Affiliations:** 1 Department of Periodontology, School of Dentistry, Aichi Gakuin University, 2-11 Suemoridori, Chikusa-ku, Nagoya 464-8651, Aichi, Japantasuku@dpc.agu.ac.jp (T.O.); tfujita2020@gmail.com (T.F.); jun1row@dpc.agu.ac.jp (J.-I.H.); minita@dpc.agu.ac.jp (A.M.); 2 Division of Molecular Genetics and Immunology, Center for Advanced Oral Science, Aichi Gakuin University, 1-100 Kusumotocho, Chikusa-ku, Nagoya 464-0037, Aichi, Japan; 3 Department of Oral Infection Medicine, Periodontology, School of Dentistry, Asahi University, 1851 Hozumi, Mizuho 501-0296, Gifu, Japan; 4 Department of Oral Pathology/Forensic Odontology, School of Dentistry, Aichi Gakuin University, 1-100 Kusumotocho, Chikusa-ku, Nagoya 464-0037, Aichi, Japan

**Keywords:** angiopoietin-like protein 2, periodontitis, *Porphyromonas gingivalis*, lipopolysaccharide, colorectal neoplasms, AOM/DSS

## Abstract

**Background/Objectives**: Recent studies suggest that angiopoietin-like protein 2 (ANGPTL2) is one of the factors contributing to disease progression in distant organs associated with periodontitis. We previously reported that periodontitis promotes hepatocellular carcinoma and that ANGPTL2 may be involved in tumor progression. Based on these findings, we herein investigated the role of periodontitis-induced ANGPTL2 in the progression of colorectal cancer (CRC) in mice. **Methods**: Male C57BL/6 mice were divided into control and periodontitis groups. Colorectal tumors were induced using azoxymethane (AOM) and dextran sulfate sodium (DSS). Periodontitis was induced by silk ligation. In addition, the model was enhanced by repeated gingival administration of lipopolysaccharide (LPS) derived from *Porphyromonas gingivalis*, a periodontal pathogen, to better mimic clinical conditions. Tumor development and ANGPTL2 expression in periodontal tissues, colorectal tumors, and serum were assessed by histology, immunostaining, and enzyme-linked immunosorbent assay. **Results**: Ligation and administration of *P. gingivalis* LPS resulted in significant alveolar bone resorption. The periodontitis group exhibited a significantly increased colorectal tumor burden compared with the control group. ANGPTL2 expression was markedly elevated in periodontal tissues, serum, and colorectal tumors in the periodontitis group. Histological analysis revealed increased tumor cell proliferation and enhanced inflammation in the periodontitis group relative to controls. These findings suggest a possible association between periodontitis-associated inflammation, elevated ANGPTL2 levels, and CRC progression in this experimental model. **Conclusions**: In this experimental model, experimental periodontitis was accompanied by concurrent increases in both local and systemic ANGPTL2 expression and accelerated growth of colorectal tumors. These findings suggest a potential association between periodontal inflammation, increased ANGPTL2 levels, and colorectal tumor progression.

## 1. Introduction

Colorectal cancer (CRC) is the second leading cause of cancer-related deaths worldwide [1]. Its etiology is multifactorial, with genetic mutations, gut microbiota, and dietary factors all implicated in its development [2]. However, the underlying mechanisms remain incompletely understood.

Periodontitis is a chronic inflammatory disease characterized by the accumulation of dental plaque, which triggers localized inflammation in the periodontal tissues [3]. When periodontal pathogens such as *Porphyromonas gingivalis* colonize periodontal pockets and invade the body, they elicit migration and activation of immune cells in response to bacterial-derived factors, including lipopolysaccharide (LPS), leading to alveolar bone destruction [4,5]. Periodontitis is not merely a localized inflammation—it is also associated with various systemic diseases, including diabetes, cardiovascular disease, and cancer [6,7,8,9,10,11]. This systemic response involves a range of inflammatory mediators, including cytokines, chemokines, prostaglandins, and matrix metalloproteinases, which exert widespread effects through the dissemination of inflammatory signals and bacterial components [3].

In recent years, poor oral hygiene has emerged as a novel lifestyle-related risk factor for CRC [12,13,14,15,16,17]. Epidemiological studies have reported that individuals with a history of periodontitis are more likely to be diagnosed with CRC, with a relative risk of 1.45 [18]. Furthermore, intervention studies suggest that periodontal treatment in patients with CRC reduces fecal *Fusobacterium nucleatum* levels, potentially preventing CRC progression [19]. Animal studies have further supported these clinical observations, demonstrating that periodontal pathogens promote colorectal carcinogenesis and accelerate CRC progression in experimental models [20,21,22]. In a mouse model of experimental periodontitis induced by silk ligation, periodontitis induction was reported to increase both the number and volume of CRC tumors, possibly through alterations in the gut and oral microbiomes [23]. However, the detailed mechanisms and mediating factors remain unclear.

Angiopoietin-like proteins (ANGPTLs) have garnered attention in diseases characterized by chronic inflammation as an underlying pathology, such as cancer. ANGPTLs are secreted proteins structurally related to angiopoietins, which are involved in angiogenesis and stem cell maintenance [24]. To date, eight types of ANGPTLs have been identified. Although they resemble angiopoietins, ANGPTLs do not bind to the classical Tie1 or Tie2 receptors [24]. While their physiological functions include angiogenesis, ANGPTLs have also been implicated in a range of biological processes, such as glucose and lipid metabolism, energy homeostasis, stem cell regulation, and tissue repair [25,26]. Among these, ANGPTL2 plays a key role in maintaining tissue homeostasis by promoting damage repair; however, because of its strong association with chronic inflammation, tissue remodeling, and cancer progression, aberrant ANGPTL2 signaling is considered a crucial molecular bridge between lifestyle-related diseases and oncogenesis [27,28,29,30]. ANGPTL2 is highly expressed in CRC tissues and has been reported to correlate with tumor size and stage [31,32,33,34,35,36,37]. Furthermore, ANGPTL2 is localized in cancer-associated fibroblasts, and some of these cells have been found to function as metastasis-promoting factors. Our group previously reported a significant increase in ANGPTL2 protein levels in the gingival crevicular fluid of patients with periodontitis. When human gingival epithelial cells were stimulated with LPS derived from *P. gingivalis*, ANGPTL2 production showed a significant increase [38]. We also demonstrated that ANGPTL2 produced in chronically inflamed periodontal tissue may contribute to the progression of hepatocellular carcinoma via the bloodstream in a mouse model of non-alcoholic steatohepatitis–hepatocellular carcinoma [39]. However, although numerous studies have suggested that the oral microbiota contributes to the association between periodontitis and CRC through the gastrointestinal tract, the role of inflammatory mediators derived from periodontal tissues in exacerbating CRC remains poorly understood, and the underlying mechanisms have not been fully elucidated.

Therefore, we hypothesized that periodontitis-associated ANGPTL2 contributes to CRC progression via systemic circulation. To test this hypothesis, we investigated the potential role of ANGPTL2 in CRC progression using a mouse model of experimental periodontal inflammation induced by silk ligature placement and local administration of LPS derived from *P. gingivalis*, a key periodontal pathogen.

## 2. Materials and Methods

### 2.1. Experimental Animals

All animal experiments were approved by the Animal Research Committee of Aichi Gakuin University (Approval No. AGUD510-2) and conducted in accordance with the Guidelines for the Care and Use of Laboratory Animals for Scientific and Ethical Reasons issued by the Science Council of Japan [40]. Four-week-old male C57BL/6 mice were purchased from Japan SLC Inc. (Shizuoka, Japan). The mice were housed at a maximum of four per cage and were allowed a 1-week conditioning period prior to the start of the experiment. They were kept in a specific pathogen-free facility under controlled environmental conditions (lights on at 8:00 a.m.; 12 h light/dark cycle) and provided with standard chow and water ad libitum. To minimize suffering, euthanasia was carried out via CO_2_ inhalation followed by cervical dislocation. Mice that experienced more than 20% body weight loss within 7 days were euthanized in accordance with humane endpoint criteria. This study adheres to the ARRIVE guidelines to ensure rigorous and ethical reporting of all animal procedures (Table S1).

### 2.2. Induction of Azoxymethane (AOM)/Dextran Sulfate Sodium (DSS)-Induced CRC in Mice

**Figure 1:**
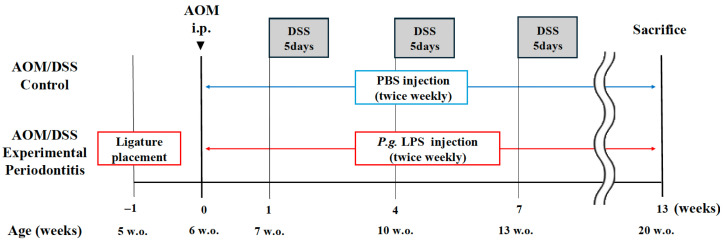
Experimental design and research schedule overview for control group mice and periodontitis group mice. AOM, azoxymethane; DSS, dextran sulfate sodium; *P.g.* LPS, *Porphyromonas gingivalis* lipopolysaccharide; PBS, phosphate-buffered saline.

An established AOM/DSS-induced CRC model was utilized as previously described (Figure 1) [41,42]. After 1 week of acclimatization, mice were randomly assigned to two groups: control (*n* = 26) and periodontitis (*n* = 27). Mice were randomly assigned to experimental groups using a computer-generated random number sequence prior to the initiation of any experimental procedures. To minimize potential bias, all outcome assessments—including alveolar bone resorption, histological evaluation, and immunohistochemical analysis—were independently performed by two investigators who were blinded to group assignments. Based on previous studies and a preliminary power analysis (G*Power 3.1.9, Düsseldorf, Germany) assuming an effect size of d = 0.8, α = 0.05, and β = 0.20 (power = 0.80), we estimated that 10–12 animals per group would be required [23,32,43]. Initial group sizes were increased to account for anticipated attrition.

At 0 week, all mice received an intraperitoneal injection of AOM (7.5 mg/kg; FUJIFILM Wako Pure Chemical Corporation, Osaka, Japan), followed by 2.0% DSS (TdB Consultancy AB, Uppsala, Sweden) in the drinking water for 5 days, and then 2 weeks of regular water. This cycle was repeated three times [44]. Body weight was recorded every 3 days. Humane endpoints were defined as difficulty eating or drinking, signs of distress (self-mutilation, abnormal posture, respiratory distress, vocalization, etc.), persistent physical abnormalities showing no signs of recovery (diarrhea, bleeding, soiled perineum, etc.), and rapid weight loss (20% or more within a few days). At 13 weeks, the mice were euthanized, and colorectal tumors were counted and measured. Tumors with a diameter of 1 mm or greater were measured using calipers. Animals that died or were euthanized prior to the scheduled endpoint were excluded from all analyses (control: *n* = 12; periodontitis: *n* = 17). Among animals surviving to 13 weeks (control: *n* = 14; periodontitis: *n* = 10), alveolar bone resorption and macroscopic tumor burden were evaluated. Tumor burden was assessed using the wild-type group without AOM/DSS administration as the baseline.

**Figure 2:**
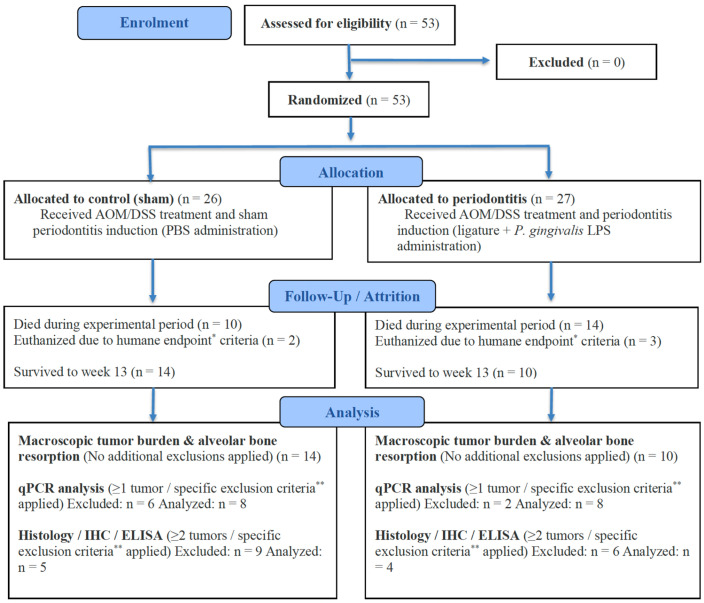
Flowchart summarizing animal allocation and reduction. * Humane endpoints (applied to macroscopic tumor burden and alveolar bone resorption): difficulty eating or drinking, signs of distress (self-mutilation, abnormal posture, respiratory distress, vocalization, etc.), persistent physical abnormalities showing no signs of recovery (diarrhea, bleeding, soiled perineum, etc.), and rapid weight loss (20% or more within a few days). ** Specific exclusion criteria (applied to qPCR and molecular/histological analyses): tooth loss, body weight loss > 20% of baseline, mortality during the experimental period, or absence of macroscopically detectable colorectal tumors at sacrifice. AOM, azoxymethane; DSS, dextran sulfate sodium; PBS, phosphate-buffered saline; LPS, lipopolysaccharide; IHC, immunohistochemical; ELISA, enzyme-linked immunosorbent assay.

Mortalities during the study were primarily due to rapid weight loss (<3 days) and were considered independent of the experimental interventions. Specific exclusion criteria were tooth loss due to progression of periodontitis, weight loss of >20%, death prior to the scheduled sacrifice, or absence of macroscopic tumors. For subsequent molecular and morphological characterization, mice with ≥1 tumor were included in quantitative polymerase chain reaction (qPCR) analyses (*n* = 8 per group), while mice with ≥2 tumors were used for histological, immunohistochemical, and enzyme-linked immunosorbent assay (ELISA) analyses (control group: *n* = 5; periodontitis group: *n* = 4) to ensure sufficient tissue availability. A flow diagram summarizing animal allocation and attrition is presented in Figure 2.

### 2.3. Induction of Experimental Periodontitis

**Figure 3:**
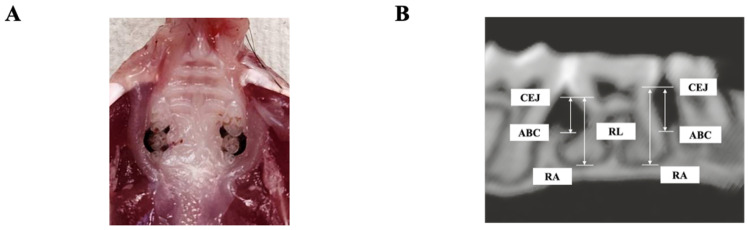
Ligation-induced experimental periodontitis and measurement points in micro-computed tomography images. Alveolar bone loss relative to root length around maxillary second molars in experimental periodontitis is shown. (**A**) Macroscopic photograph showing silk ligature around the cervical region of the maxillary second molar. (**B**) Method for analyzing alveolar bone resorption in micro-computed tomography images. The ratio of residual alveolar ridge bone to root length was calculated using the formula (ABC − RA)/RL, where (ABC − RA) denotes the distance from the ABC to the RA, and RL denotes the distance from the CEJ to the RA. CEJ, cementoenamel junction; ABC, alveolar bone crest; RA, root apex; RL, root length.

In the periodontitis group, as previously described [45], 5-0 silk sutures were ligated around both maxillary second molars at 5 weeks of age (Figure 3A). To mimic persistent mechanical plaque retention, the ligatures were maintained from 1 week prior to AOM administration until sacrifice (approximately 14 weeks) without routine replacement. Sutures were inspected twice weekly, and if detachment due to suture loosening or material degradation was observed, the ligatures were re-ligated. No significant tissue damage or foreign body reaction was observed during the prolonged ligation period.

During the DSS treatment period, to better mimic clinical conditions, 10 µL of *P. gingivalis*-derived LPS (2.0 µg/µL; InvivoGen, San Diego, CA, USA) was injected into the gingiva surrounding both maxillary second molars using a microsyringe (total dose: 20 µg) to enhance the periodontitis model [46,47]. The control group received phosphate-buffered saline injected into the same site. Gingival LPS injections were performed twice weekly for 13 weeks, starting immediately after AOM administration. Anesthesia was induced using a combination of medetomidine, midazolam, and butorphanol [48].

### 2.4. Micro-Computed Tomography Analysis

Alveolar bone resorption was evaluated at the start of ligation and at the time of sacrifice using micro-computed tomography (Rigaku, Tokyo, Japan) in accordance with the standard protocol [49]. Bone resorption was measured and analyzed according to the method described by Izawa et al. (Ratoc, Tokyo, Japan) [50], using root length as a reference, by measuring the distance from the cementoenamel junction to the alveolar bone crest (Figure 3B). Measurements were performed by two independent researchers under blinded conditions.

### 2.5. Real-Time PCR Analysis

qPCR analysis was performed as previously described (Thermo Fisher Scientific, Wilmington, DE, USA). Total RNA was isolated from gingival and tumor tissues (Macherey-Nagel, Düren, Germany). RNA quality was assessed by measuring the A260/A280 nm ratio using a fluorometer (Thermo Fisher Scientific). qPCR was performed using TaqMan Universal PCR Master Mix with Angptl2 (Mm00507897_m1), Tnf (Mm00443258_m1), and Il1b (Mm00434228_m1) as target genes. Actb (Mm02619580_g1) was used as the endogenous control (Thermo Fisher Scientific), and gene expression levels were calculated using the 2^−ΔΔCt^ method [51].

### 2.6. Histological Analysis

Maxillary bone and colon tissues were rinsed with phosphate-buffered saline and fixed in 4% paraformaldehyde (maxillary bone: 7 days; colon tissue: 3 days) [52]. Maxillae were decalcified in 10% EDTA at 4 °C for 7 days. Tissues were then paraffin-embedded, sectioned into 4 μm slices, and stained with hematoxylin and eosin (Muto Pure Chemicals, Tokyo, Japan). Sections were examined by light microscopy (BX51; Olympus, Tokyo, Japan) [53]. Histological evaluation was performed independently by two investigators under blinded conditions. Tumor morphology was assessed in hematoxylin and eosin–stained sections, based on findings such as glandular architecture, epithelial atypia, inflammatory cell infiltration, mitosis, and the presence of necrotic areas. The mitotic figures were systematically compared between groups to identify differences in tumor morphology.

### 2.7. Immunohistochemical Analysis

Immunohistochemistry was performed using anti-ANGPTL2 antibody (Proteintech Group, Chicago, IL, USA) and anti-Ki67 antibody (Abcam, Waltham, MA, USA) [54]. Quantification of positive cells was performed by two independent investigators who were blinded to the group allocation. Positive cells were counted in three fields (each measuring 1 × 10^4^ μm^2^) at 400× magnification by two blinded investigators. Cells showing cytoplasmic ANGPTL2 staining were counted as ANGPTL2-positive. For Ki67, cells exhibiting nuclear staining were counted as positive. The degree of alveolar bone resorption, tumor measurements, and immunohistochemical cell counting were assessed by investigators blinded to the group allocation.

### 2.8. ELISA

Serum ANGPTL2 levels were measured using a commercial ELISA kit (Elabscience Biotechnology, Houston, TX, USA) with serum samples collected at 14 weeks, following the manufacturer’s instructions.

### 2.9. Statistical Analysis

The primary endpoint was total tumor burden (i.e., number of colorectal tumors) at 13 weeks. Key secondary endpoints were ANGPTL2 expression levels in the colon and related inflammatory markers. Survival rate, body weight change, tumor size distribution, and alveolar bone resorption were analyzed as secondary outcomes. qPCR and ELISA analyses were considered exploratory.

Normality of the data was assessed using the Shapiro–Wilk test. When data were not normally distributed, they were analyzed using the Mann–Whitney U test and are presented as median and interquartile range (IQR). When data were normally distributed, they were analyzed using the unpaired *t*-test and are presented as mean ± standard deviation (SD). Most intergroup comparisons, including alveolar bone resorption, tumor number, tumor size, gene expression levels, the number of positive cells in immunohistochemical staining, and ELISA measurements, were analyzed using the Mann–Whitney U test. Survival rates were analyzed using the log-rank test. Body weight changes over time were analyzed using two-way analysis of variance followed by Bonferroni’s multiple-comparison test. The number of tumors categorized by maximum diameter was analyzed using an unpaired multiple *t*-test. Where multiple comparisons were performed, Bonferroni correction was applied to control for type I error.

The correlation between ANGPTL2 levels and tumor burden was assessed using Spearman’s rank correlation coefficient. Simple linear regression analysis was performed to evaluate the association between ANGPTL2 expression and tumor number. A *p* value of <0.05 was considered statistically significant. Ninety-five percent confidence intervals were calculated where appropriate. All statistical analyses were performed using GraphPad Prism 6.0g (GraphPad Software, Boston, MA, USA) [55].

## 3. Results

### 3.1. Experimental Periodontitis Promotes the Growth of Colorectal Tumors in AOM/DSS Mice

**Figure 4:**
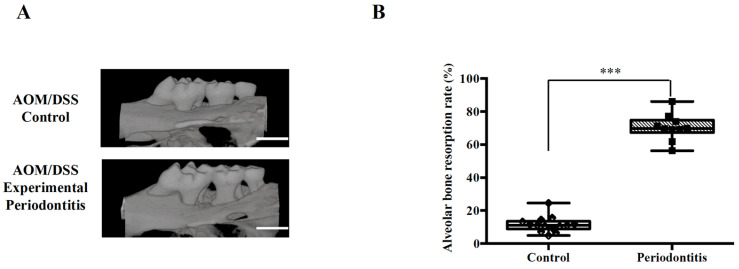
Alveolar bone resorption rate in experimental periodontitis. (**A**) Comparison of jawbones between groups with and without periodontitis in AOM/DSS mice. Representative images obtained by micro-computed tomography are shown. Scale bar: 1000 µm. (**B**) Ratio of residual alveolar bone to root length (control: *n* = 14; periodontitis: *n* = 10). Intergroup differences were analyzed using the Mann–Whitney U test. Data are presented as median and interquartile range. *** *p* < 0.001. AOM, azoxymethane; DSS, dextran sulfate sodium.

**Figure 5:**
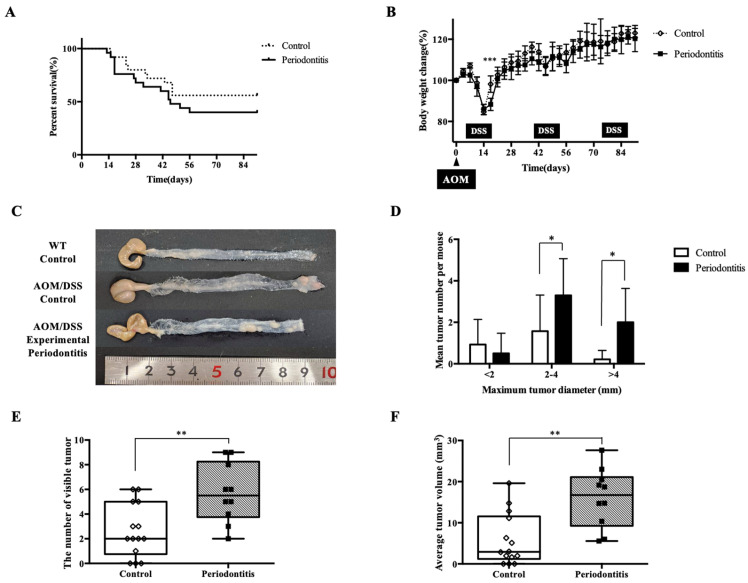
Survival, body weight changes, and colorectal tumor formation in AOM/DSS mice. (**A**) Survival rate and (**B**) body weight change during the experimental period (control: *n* = 26; periodontitis: *n* = 27). (**C**) Representative macroscopic images of the colon at the time of sampling in WT, control, and periodontitis groups (control: *n* = 14; periodontitis: *n* = 10). The WT group did not receive AOM/DSS treatment. (**D**) Number of tumors according to size. (**E**,**F**) Total number and volume of visible tumors in the colon of each group (control: *n* = 14; periodontitis: *n* = 10). Intergroup differences were analyzed using the log-rank test in (**A**), two-way analysis of variance followed by Bonferroni’s multiple-comparison test in (**B**), unpaired multiple *t*-tests in (**D**), and the Mann–Whitney U test in (**E**,**F**). Data are presented as median (IQR) or mean ± SD, as appropriate. * *p* < 0.05, ** *p* < 0.01, *** *p* < 0.001. AOM, azoxymethane; DSS, dextran sulfate sodium; WT, wild type.

To investigate the effect of experimental periodontitis on colorectal tumor development, tumors were induced in mice using the AOM/DSS model (Figure 1). As shown in Figure 4A, significant periodontal tissue destruction was confirmed in mice subjected to experimental periodontitis induced by silk ligation and LPS administration. In the periodontitis group, alveolar bone resorption was significantly increased compared with the control group (median [IQR]: 11.14 [8.893–13.57] in the control group vs. 69.76 [67.29–74.77] in the periodontitis group, *p* < 0.001) (Figure 4B). Twenty-six control group mice and twenty-seven periodontitis group mice were administered AOM at 0 week. At the time of sacrifice (13 weeks), 14 mice in the control group and 10 mice in the periodontitis group had survived (*p* = 0.3686) (Figure 5A).

Continuous monitoring of body weight throughout the experimental period revealed no significant differences between groups; however, recovery from first DSS-induced weight loss was significantly slower in the periodontitis group than in the control group (*p* < 0.001) (Figure 5B). Transverse colon sections and macroscopic examination of the mucosal surface showed that the total number of tumors (2.00 [0.75–5.00] in the control group vs. 5.50 [3.75–8.25] in the periodontitis group) and tumor size (2.926 [1.223–11.57] mm^3^ in the control group vs. 16.74 [9.272–21.12] mm^3^ in the periodontitis group) were significantly larger in the periodontitis group (*p* < 0.01) (Figure 5C,E,F). Furthermore, the number of tumors with a maximum diameter of 2 mm or more was significantly higher in the periodontitis group (*p* < 0.05) (Figure 5D).

### 3.2. Experimental Periodontitis Significantly Increases ANGPTL2 Expression in Periodontal Tissue

**Figure 6:**
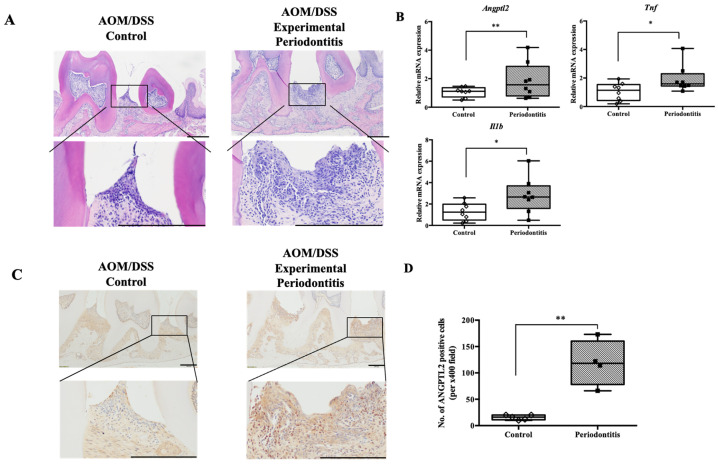
Histological analysis of periodontal tissue and ANGPTL2 production. (**A**) Representative hematoxylin- and eosin-stained sections from mice with and without periodontitis (scale bar: 100 µm; magnifications ×100 and ×400). (**B**) mRNA expression of ANGPTL2 and inflammatory cytokines (TNF-α, IL-1β) in gingival tissue detected by qPCR (*n* = 8/group). (**C**) Immunohistochemical images using an ANGPTL2 antibody in mice with and without periodontitis (scale bar: 100 µm; magnifications ×100 and ×400). (**D**) Comparison of the number of ANGPTL2-positive cells in the periodontal tissues of control and periodontitis mice (control: *n* = 5; periodontitis: *n* = 4). Intergroup differences were analyzed using the Mann–Whitney U test. Data are presented as median and interquartile range (IQR). * *p* < 0.05, ** *p* < 0.01. AOM, azoxymethane; DSS, dextran sulfate sodium; ANGPTL2, angiopoietin-like protein 2.

Periodontal tissue from mice with experimentally induced periodontitis (ligation combined with LPS administration) showed elongation of epithelial projections and infiltration of inflammatory cells into both the epithelial and subepithelial layers (Figure 6A). mRNA analysis of gingival tissue revealed significantly increased expression of Angptl2 (control group: 1.118 [0.7189–1.367] vs. periodontitis group: 1.571 [0.7925–2.862], *p* < 0.01), Tnf (1.141 [0.4297–1.543] vs. 1.595 [1.440–2.299], *p* < 0.05), and Il1b (1.242 [0.5017–1.985] vs. 2.655 [1.589–3.695], *p* < 0.05) in the periodontitis group compared with the control group (Figure 6B). Immunohistochemical staining revealed a significant increase in ANGPTL2-positive cells in the periodontal tissues of the periodontitis group compared with the control group (median [IQR]: 16.0 [11.0–20.0] cells in the control group vs. 118.0 [78.0–160.3] cells in the periodontitis group, *p* < 0.01) (Figure 6C,D).

### 3.3. Experimental Periodontitis Promotes the Progression of Colorectal Tumors

**Figure 7:**
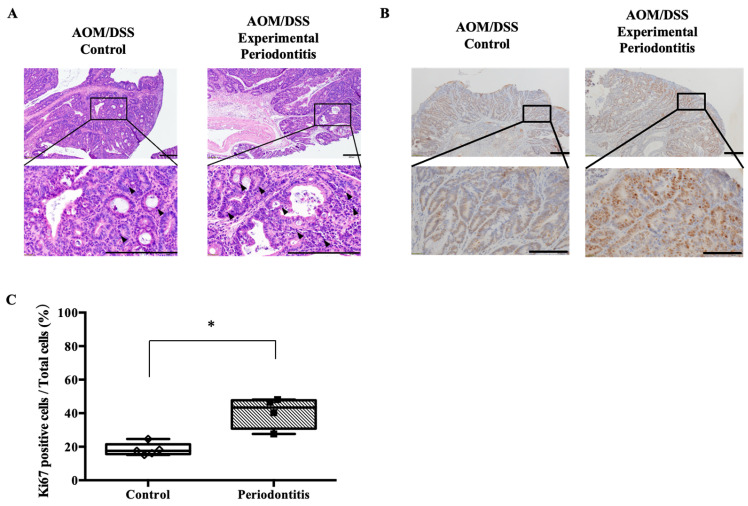
Histological analysis of colorectal tumors. (**A**,**B**) Representative hematoxylin- and eosin-stained and immunohistochemical images of colorectal tumors (scale bar: 200 µm; magnifications ×100 and ×400). The arrowhead indicates mitosis. (**C**) Comparison of Ki67-positive cell percentage (control: *n* = 5; periodontitis: *n* = 4). Intergroup differences were analyzed using the Mann–Whitney U test. Data are presented as median and interquartile range (IQR). * *p* < 0.05. AOM, azoxymethane; DSS, dextran sulfate sodium.

To further investigate tumor pathology, histopathological analysis of colorectal tumors was performed. Hematoxylin and eosin staining showed typical features of colorectal adenocarcinoma, including loss of epithelial polarity, an increased nuclear-to-cytoplasmic ratio, and inflammatory cell infiltration. These histopathological features, which were qualitatively evaluated by two investigators blinded to the group allocation, are commonly associated with increased tumor aggressiveness. Tumors in the periodontitis group tended to exhibit increased cellular density, nuclear hyperchromasia, and numerous mitotic figures compared with those in the control group (Figure 7A). Furthermore, tumors in the periodontitis group tended to show a higher number of mitotic figures than those in the control group (control: 6.0 [4.5–10.0] vs. periodontitis: 10.5 [10.0–11.75], *p* = 0.0635). No apparent differences in histologic tumor grade or differentiation were observed between the groups. Immunohistochemical staining for the proliferation marker Ki67 demonstrated a significantly higher positive cell rate in the periodontitis group than in the control group (control group: 17.58 [15.63–21.45] vs. periodontitis group: 43.41 [30.81–47.73], *p* < 0.05) (Figure 7B,C).

### 3.4. Expression of ANGPTL2 in Colorectal Tumors in AOM/DSS Mice with Experimental Periodontitis Was Significantly Increased

**Figure 8:**
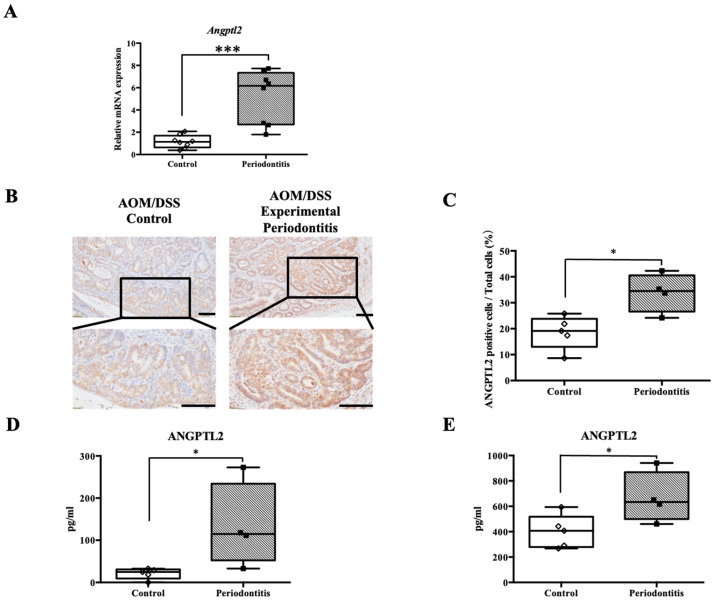
Analysis of ANGPTL2 protein in colorectal tumors and serum. (**A**) mRNA expression levels of ANGPTL2 in colorectal tumors by qPCR (*n* = 8/group). (**B**) ANGPTL2 expression in colorectal tumors from control and periodontitis groups in AOM/DSS mice (scale bar: 200 µm; magnifications ×100 and ×400). (**C**) Number of ANGPTL2-positive cells in colorectal tumor tissue. (**D**) ANGPTL2 levels in colorectal tumors. (**E**) ANGPTL2 concentration in serum from AOM/DSS mice (control: *n* = 5; periodontitis: *n* = 4). Intergroup differences were analyzed using the Mann–Whitney U test. Data are presented as median and interquartile range (IQR). * *p* < 0.05, *** *p* < 0.001. AOM, azoxymethane; DSS, dextran sulfate sodium; ANGPTL2, angiopoietin-like protein 2.

To investigate potential factors contributing to tumor progression in mice with experimental periodontitis, we focused on ANGPTL2 and evaluated its expression in colorectal tumors. mRNA analysis of colorectal tumors revealed significantly higher ANGPTL2 mRNA expression in the periodontitis group than in the control group (control group: 1.141 [0.6360–1.696] vs. periodontitis group: 6.168 [2.692–7.349], *p* < 0.001) (Figure 8A). Immunohistochemical analysis revealed a significant increase in ANGPTL2-positive cells in colorectal tumors from the periodontitis group compared with the control group (19.15 [13.03–23.82] vs. 34.52 [26.60–40.57], *p* < 0.05) (Figure 8B,C). ANGPTL2 levels in colorectal tumors from AOM/DSS mice with periodontitis were also significantly higher than those in the control group, as determined by ELISA using tumor cell suspensions (24.89 [9.291–30.89] pg/mL in the control group vs. 114.8 [52.48–234.1] pg/mL in the periodontitis group, *p* < 0.05) (Figure 8D).

We previously demonstrated that stimulating human gingival epithelial cells with recombinant ANGPTL2 significantly increases ANGPTL2 production [37]. This increase in ANGPTL2 within colorectal tumors suggests that hematogenously derived ANGPTL2 from periodontal tissues may influence colorectal epithelial cells. To explore potential systemic effects of periodontitis on colorectal tumor progression, serum ANGPTL2 levels were measured. Serum ANGPTL2 levels in the periodontitis group (633.8 [499.8–868.8] pg/mL) were significantly elevated compared with those in the control group (407.3 [279.1–517.7] pg/mL) (*p* < 0.05) (Figure 8E).

ANGPTL2 expression in colorectal tumors was positively correlated with tumor number (Spearman’s r = 0.62, *p* = 0.004). Simple linear regression analysis confirmed that ANGPTL2 expression significantly predicted tumor burden (β = 0.55, *p* = 0.01).

## 4. Discussion

In this study, we found that experimental periodontitis induced by ligation and LPS administration in AOM/DSS-induced CRC model mice was associated with a significant increase in colorectal tumor burden and in the expression of the proliferation marker Ki67 within tumor tissue. When experimental periodontitis was induced, increased ANGPTL2 expression occurred simultaneously in both colorectal tumor tissue and the systemic circulation, suggesting a possible association between elevated ANGPTL2 levels and CRC progression.

ANGPTL2 is a multifunctional secreted protein that promotes chronic inflammation, angiogenesis, and worsening of the tumor microenvironment, thereby contributing to cancer progression [30,31,32,33,34,35,36,37,39]. In this study, we observed a significant increase in ANGPTL2 expression in the periodontal tissues of CRC mice with experimentally induced periodontitis. Experimental periodontitis models are typically examined over short durations (1–2 weeks); however, in this study, we aimed to induce a sustained systemic inflammatory response associated with periodontitis. To achieve this, ligature placement was maintained for an extended period (14 weeks). The inflammatory mechanism of experimental periodontitis typically involves epithelial destruction by day 1, a marked increase in anaerobic bacteria by day 5, and a peak in inflammatory cytokines around day 7. Subsequently, the epithelium enters a repair phase, during which cytokine expression is thought to be suppressed [56].

In this study, we generated CRC mice with experimentally induced periodontitis via ligation (ligature group) or combined ligation and administration of periodontal pathogen LPS (ligature + LPS group). Ligation and injection procedures were performed under anesthesia, with *P. gingivalis* LPS injections administered twice weekly for 13 weeks. No adverse events were observed in the mice throughout the experimental period. Comparing the ligation group and the ligature + LPS group, mRNA expression of inflammatory cytokines in gingival tissue was significantly elevated in the latter group (Figure S1). However, no significant changes in ANGPTL2 mRNA expression were observed in colorectal tumors in either group (Figure S2). Regarding tumor formation, the number of tumors reaching a specific size (≥2 mm and <4 mm) was significantly increased in the ligature + LPS group (Figure S3). These findings suggest that maintenance of a chronic inflammatory state through continuous *P. gingivalis* LPS administration together with ligation might be associated with increased ANGPTL2 expression in periodontal tissues, which may be associated with tumor formation in this experimental model. Furthermore, the increase in medium-sized tumors (maximum diameter of ≥2 mm and <4 mm) associated with *P. gingivalis* LPS administration to periodontal tissue may reflect not only chronic inflammation but also influences from other tissues. These results suggest that increased ANGPTL2 production associated with periodontitis may enter systemic circulation and affect other organs via the bloodstream. However, because this model combines ligature placement with repeated gingival administration of *P. gingivalis*-derived LPS, it does not conclusively demonstrate that periodontal tissue-derived ANGPTL2 is the sole causal mediator. The inflammatory conditions generated in this experimental system therefore represent a composite inflammatory exposure and may not fully recapitulate the pathophysiology of naturally occurring periodontitis. In our previous report, we showed that recombinant ANGPTL2 increases ANGPTL2 expression in epithelial cells, indicating an autocrine response [37]. Upon stimulation of the human gingival epithelial cell line Ca9-22 with *P. gingivalis* LPS, ANGPTL2 gene expression was significantly upregulated (Figure S4). Furthermore, conditioned medium collected from *P. gingivalis* LPS-stimulated Ca9-22 cells significantly enhanced the proliferation of the human CRC cell line HCT-116 (Figure S5). We further observed elevated ANGPTL2 expression in colorectal tumor tissue, suggesting that ANGPTL2 derived from periodontal tissue may circulate systemically and might be associated with increased ANGPTL2 expression within colorectal tumors. In addition, we previously reported that ANGPTL2 may promote hepatocellular carcinoma progression via systemic circulation in STAM mice [38].

The hypothesis that ANGPTL2 induced by periodontitis may circulate systemically and modulate the microenvironment of distant organs, including colorectal tumors, provides a potential biological explanation for the observed association within this experimental model combining ligature placement and repeated *P. gingivalis* LPS exposure. In the present study, both tissue and serum ANGPTL2 levels were elevated in the periodontitis group and were positively correlated with tumor burden, supporting a possible contributory role of ANGPTL2 in periodontitis-associated CRC progression, although causality cannot be inferred from this study alone.

ANGPTL2 has been reported to promote chronic inflammation through activation of integrin-mediated signaling pathways and downstream NF-κB activation [57,58], thereby enhancing the expression of pro-inflammatory cytokines and facilitating tumor progression. Consistent with our findings, Toiyama et al. [59] reported that ANGPTL2 is overexpressed in CRC tissue and is significantly associated with advanced T stage, lymph node metastasis, and liver metastasis. Furthermore, elevated serum ANGPTL2 levels in patients with CRC have been linked to early relapse and poor prognosis [35,59]. Taken together, these findings suggest a potential association between periodontitis-associated systemic inflammation, increased ANGPTL2 levels, and CRC progression.

However, this study has several limitations. Although the association between periodontitis and CRC has been previously reported, with multiple studies suggesting that oral bacteria may disrupt the intestinal microbiota via the digestive tract or bloodstream (potentially promoting CRC progression [20,60,61,62]), this study did not include an analysis of the intestinal microflora. Such analyses will be necessary in future investigations to clarify the contribution of microbial dysbiosis to the observed tumor-promoting effects. A further limitation is the exclusive use of the AOM/DSS model, which primarily reflects inflammation-associated colorectal carcinogenesis and does not encompass the full spectrum of human CRC. DSS induces acute chemical colitis through epithelial injury and therefore does not fully recapitulate the chronic, immune-mediated, and relapsing–remitting features of human inflammatory bowel disease. Accordingly, the tumor-promoting effects observed in the present study may partly reflect exacerbation of chemically induced mucosal inflammation rather than direct modulation of tumor-initiating pathways. Moreover, AOM-driven mutagenesis represents a restricted molecular route of carcinogenesis and does not capture the heterogeneity of sporadic CRC. Caution is therefore warranted when extrapolating these findings to broader clinical contexts. Validation using genetic or spontaneous tumor models will be necessary to distinguish inflammation-dependent effects from tumor-intrinsic mechanisms.

Additionally, it remains unclear whether the elevated ANGPTL2 levels detected in serum and tumor tissues are truly attributable to periodontal tissue; the contribution of both periodontitis- and colorectal tumor-derived factors should be considered. Future mechanistic studies are required to determine whether administration of exogenous ANGPTL2 to mice without periodontitis would reproduce the tumor-promoting effects observed in the periodontitis group.

## 5. Conclusions

In an AOM/DSS-induced mouse model, ligation- and LPS-induced experimental periodontitis was associated with an increased number and volume of CRC tumors and with increased local and systemic ANGPTL2 levels. These findings suggest a potential association between elevated ANGPTL2 levels and the relationship between oral inflammation and CRC progression. However, the current data are only associational and do not establish a causal relationship. Further research, including functional validation experiments using genetic or pharmacologic ANGPTL2 inhibition, as well as comprehensive microbiome profiling, is essential to clarify these relationships and to support the development of effective prevention strategies.

## Data Availability

The data presented in this study are not publicly available because of institutional restrictions but are available from the corresponding author upon reasonable request.

## Supplementary Materials

The following supporting information can be downloaded at: https://www.mdpi.com/article/10.3390/jcm15062359/s1, Figure S1: mRNA expression levels of inflammatory cytokines in gingival tissue. Figure S2: mRNA expression levels in colorectal tumors by qPCR. Figure S3: Measurement of colorectal tumors in CRC mice. Figure S4: *Porphyromonas gingivalis* LPS stimulation significantly increased ANGPTL2 mRNA in Ca9-22 cells (qPCR). Figure S5: Treatment of HCT116 cells with the culture supernatant of *P. gingivalis* LPS-stimulated Ca9-22 cells promoted cell proliferation, as measured by the MTT assay. Table S1: The ARRIVE guidelines 2.0: author checklist.

## Abbreviations

The following abbreviations are used in the main text of this manuscript:

ANGPTL2	angiopoietin-like protein 2
CRC	colorectal cancer
AOM	azoxymethane
DSS	dextran sulfate sodium
LPS	lipopolysaccharide
ELISA	enzyme-linked immunosorbent assay
qPCR	quantitative polymerase chain reaction
IQR	interquartile range
SD	standard deviation
ABC	alveolar bone crest
RA	root apex
RL	root length
