# Supplementary material for: Increased Production of Angiopoietin-like Protein 2 in a Ligature- and LPS-Induced Periodontitis Mouse Model May Promote Colorectal Tumor Progression
Source: J Clin Med. 2026 Mar 19;15(6):2359. doi: 10.3390/jcm15062359 (PMC13026960; doi:10.3390/jcm15062359)
Supplement: Supplementary file 1 [file jcm-15-02359-s001.zip › Supplementary Figures.pdf]

Supplementary Figure S1.

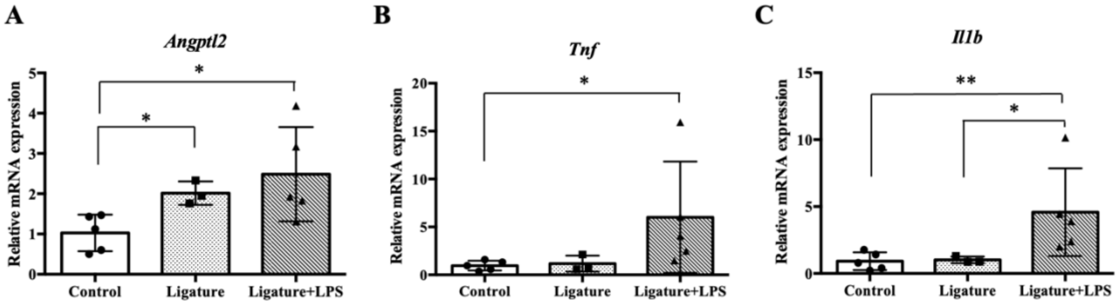

Figure S1. mRNA expression levels of inflammatory cytokines in gingival tissue. (A, B, C) Relative mRNA expression of ANGPTL2, TNF-α, and IL-1β. Control group n = 5, ligation group n = 3, ligation + LPS group n = 5. *$p < 0.05$, **$p < 0.01$.

Supplementary Figure S2.

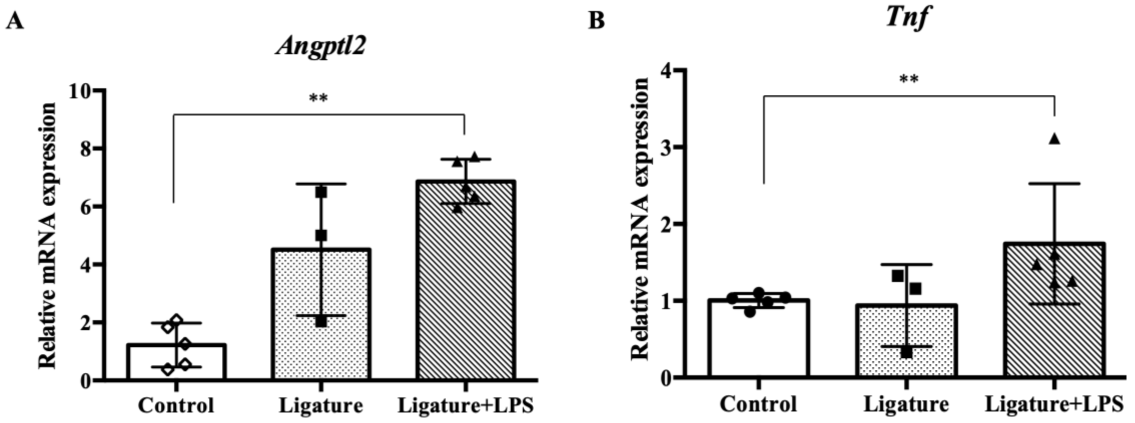

Figure S2. mRNA expression levels in colon tumors by qPCR. (A, B) Relative mRNA expression of ANGPTL2 and TNF-α. Control group n = 5, ligation group n = 3, ligation + LPS group n = 5. ***p* < 0.01.

Supplementary Figure S3.

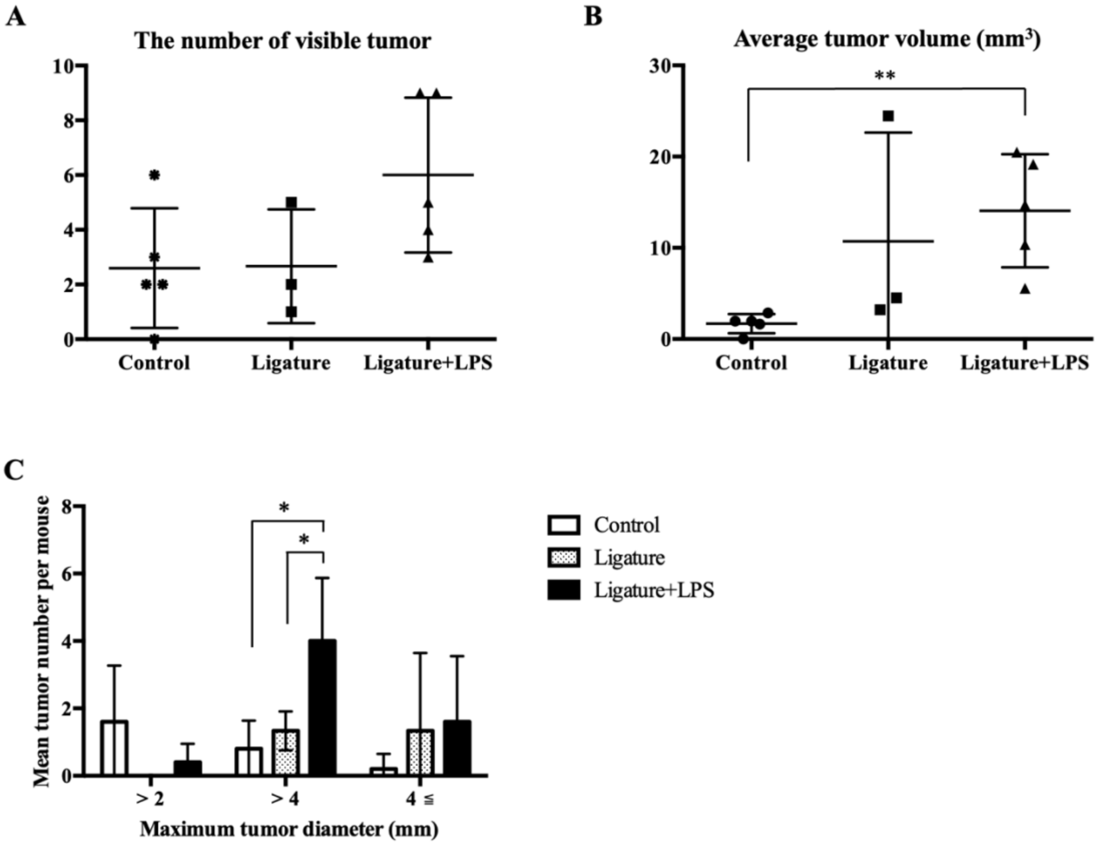

Figure S3. Measurement of colorectal tumors in CRC mice. (A, B) Total number of tumors and tumor volume in the colon. (C) Number of tumors per size category. Control group n = 5 Ligature group n = 3, Ligature + LPS group n = 5. *$p < 0.05$, **$p < 0.01$.

Supplementary Figure S4.

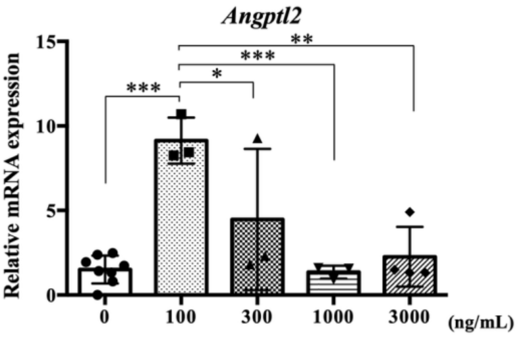

Figure S4. *Porphyromonas gingivalis* LPS stimulation significantly increased ANGPTL2 mRNA in Ca9-22 cells (qPCR).

Induction of ANGPTL2 mRNA expression in Ca9-22 cells pre-stimulated with IFN-γ and subsequently treated with various concentrations of *P. gingivalis* LPS. Ca9-22 gingival epithelial cells were pre-stimulated with IFN-γ overnight and then treated for 24 hours with *P. gingivalis* LPS at concentrations of 0, 100, 300, 1000, or 3000 ng/mL. Total RNA was extracted, and ANGPTL2 mRNA expression was quantified by qPCR. Data were presented as mean ± SD (n = 3 or 4 or 8). *$p < 0.01$. **$p < 0.01$. ***$p < 0.001$. Statistical significance assessed by Paired t test.

Supplementary Figure S5.

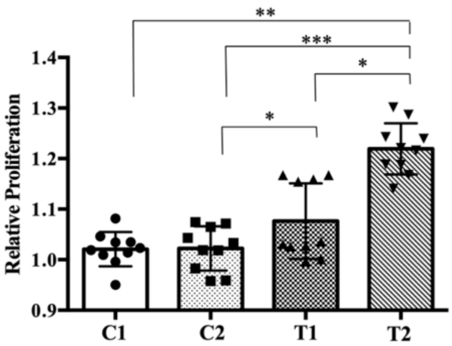

Figure S5. Treatment of HCT116 cells with the culture supernatant of *P.gingivalis* LPS stimulated Ca9-22 cells promoted cell proliferation as measured by the MTT assay.

Effects of conditioned medium from P. gingivalis LPS–stimulated Ca9-22 cells on the proliferation of HCT-116 cells assessed by the MTT assay. Data were presented as mean ± SD (n = 10). *p < 0.01. **p < 0.01. ***p < 0.001. C1, DMEM alone (no stimulation); C2, DMEM containing *P. gingivalis* LPS (100 ng/mL)); T1, Culture supernatant of Ca9-22 cells cultured in DMEM without *P. gingivalis* LPS; T2, Culture supernatant of Ca9-22 cells cultured containing *P. gingivalis* LPS (100 ng/mL). Statistical significance assessed by Paired t test.
